# The Cish SH2 domain is essential for PLC-γ1 regulation in TCR stimulated CD8^+^ T cells

**DOI:** 10.1038/s41598-018-23549-2

**Published:** 2018-03-28

**Authors:** Geoffrey Guittard, Ana Dios-Esponera, Douglas C. Palmer, Itoro Akpan, Valarie A. Barr, Asit Manna, Nicholas P. Restifo, Lawrence E. Samelson

**Affiliations:** 1 Laboratory of Cellular and Molecular Biology, Center for Cancer Research, NCI, National Institutes of Health, Bethesda, Maryland 20892-4256 USA; 2 Surgery Branch, Center for Cancer Research, National Cancer Institute, National Institutes of Health, 9000 Rockville Pike, Building 10/CRC, Room 3W-3840, Bethesda, MD 20892 USA; 3 Present Address: INSERM, U1068, CNRS UMR7258, Aix-Marseille Université UM105, Institut Paoli-Calmettes, Centre de Recherche en Cancérologie de Marseille, 13009 Marseille, France

## Abstract

Cish, participates within a multi-molecular E3 ubiquitin ligase complex, which ubiquitinates target proteins. It has an inhibitory effect on T cell activation mediated by PLC-γ1 regulation, and it functions as a potent checkpoint in CD8^+^ T cell tumor immunotherapy. To study the structural and functional relationships between Cish and PLC-γ1 during CD8^+^ T cell activation, we tested mutants of the Cish-SH2 (R107K) and D/BC (L222Q, C226Q) domains. We confirmed that Cish-SH2-specific binding was essential for PLC-γ1 ubiquitination and degradation. This domain was essential for the Cish-mediated inhibition of Ca^2+^ release upon TCR stimulation. No effect on inhibition of cytokine release was observed with SH2 or D/BC mutants, although the absence of Cish led to an increased release of IFN-γ and TNF-α. Using imaging we showed that Cish was expressed mostly in the cytoplasm and we did not see any Cish clustering at the plasma membrane upon stimulation. We conclude that the Cish-SH2 domain is essential for PLC-γ1 regulation in TCR-stimulated CD8^+^ T cells.

## Introduction

Cish (cytokine-inducible SH2 containing protein) belongs to the SOCS (Suppressor of Cytokine signaling) family of E3-ligases. Emerging evidence indicates that SOCS family members can play critical roles in both innate and adaptive immune responses by mediating negative-feedback inhibition of cytokine signaling^1^. Lack of Cish expression in cytotoxic CD8^+^ T cells and NK cells improves their anti-tumor properties^2–4^. Understanding the precise molecular mechanisms for Cish function is thus of great interest and might suggest possibilities for new targeted cancer therapies.

We previously observed that Cish is expressed following T cell antigen receptor (TCR) stimulation^2^. We also showed that Cish is involved in negative regulation of TCR signaling. Cish can bind PLC-γ1 constitutively and negatively regulates its phosphorylation and activation. This interaction leads to PLC-γ1 ubiquitination and subsequent degradation. Thus our data identified Cish as a new key negative regulator of TCR signaling and immune function^2^.

There are eight SOCS family members, SOCS1–7 and Cish. Each of these proteins has a conserved structure with a central Src homology 2 (SH2) domain, an amino-terminal domain of variable length and a carboxy-terminal 40-amino-acid module known as the SOCS box. The SOCS box interacts with several ubiquitination machinery enzymes: elongin B/C, cullins, ring-box 2 (Rbx2) and an E2 ubiquitin transferase^1^. This complex forms an E3 ubiquitin ligase complex. Thus SOCS proteins exert their inhibitory role by mediating protein degradation. The central SH2 domain appears critical for binding target molecules, thereby enabling the E2-E3 complex to ubiquitinate them^5^. Cish was the first identified member of the SOCS family. It was discovered as a gene product induced in response to various cytokines (IL-2, 3, 5 and EPO) that activate STAT5^6,7^. Cish was shown to bind via its SH2 domain to cytokine receptors after ligand-mediated phosphorylation^8,9^. Cish can act as a negative-feedback regulator of the STAT5 pathway by binding in this manner, thereby masking STAT5 docking sites^9,10^.

In order to define the structural and functional relationships between Cish and PLC-γ1 during CD8^+^ T cell activation, we used both SH2 domain (Cish SH2*) and D/BC SOCS box (Cish D/BC*) Cish mutants to check for their impact on PLC-γ1 and CD8^+^ T cell function. Underlying our study is our desire to identify a dominant negative mutant of Cish, which would be of great interest to understand more precisely the regulation of Cish and might give some insight into approaches that would specifically target Cish for inhibition.

## Materials and Methods

### Cells and culture

293 T Cells were maintained in Dulbecco’s modified Eagle’s medium (DMEM) supplemented with 10% fetal bovine serum, and 1% penicillin-streptomycin. Cells were incubated at 37 °C in 5% CO_2_.

E6-1 Jurkat cell lines were cultured in RPMI supplemented with 10% fetal bovine serum and 1% penicillin–streptomycin.

### Mice

*Cish*^−/−^ mice were generated and genotyped, as previously described^2^. Mice were housed according to the guidelines of the Animal Care and Use Committee at the National Institutes of Health (NIH).

### Transfection 293 T cells

293 T cells were transfected using a calcium-phosphate transfection method. Briefly, for transfection, 2.5 × 10^6^ cells were plated 24 hrs prior to transfection in a 10-cm dish. On the day of transfection, a 500 μL aqueous mixture of DNA (5 μg max per construct plus pcDNA3 empty vector, to reach 20 μg total DNA per dish) and CaCl_2_ (62 μl of 2M CaCl_2_) was added dropwise to 500 μL of 2X HBS (42 mM HEPES, 274 mM Nacl, 10 mM KCL, 1.8 mM Na_2_PO_4_) (pH 6.95–7.00). 10 mL of fresh medium was then added to the 293 T cells, and the HBS/DNA mixture was added dropwise to the cells. 24 hrs later the medium was replaced with 10 mL of fresh media.

### Cloning

The Cish WT expression retroviral vector has already been described^2^ and encodes N-terminal Flag-tagged (3×) Cish, self-cleaving furin-2A (f2A) peptide, and congenic marker Thy1.1 driven by the intrinsic LTR promoter. We generated the SH2 domain R107K^10^ and the SOCS-Box D/BC (L222Q, C226Q) mutations^11^ in the same backbone vector (Genesynthesis Company). Cish and Cish mutant constructs were expressed in pmTurquoise2-C1 (Addgene #60560^12^) vectors (Kind gift of Dorus Gadella). In-fusion kit from Clontech was used and cloning was performed according to manufacturer’s instructions.

### Transfection of Jurkat cells, fixation and immunostaining

Jurkat T cells were transfected using the Amaxa T-kit. Cells were allowed to spread on coverslips as described earlier^13^. Briefly, poly-lysine covered four-chambered glass coverslips (LabTek II, Nunc/Nalgene) were coated with 10 μg/ml of antibody (anti-CD3-Ucht1) from BD Pharmigen (custom concentration of Catalog # 550367). The chambers were loaded with 300 μl of normal media without phenol red supplemented with 25 mM HEPES, pH 7.0, and warmed. Cells were resuspended in the same buffer, plated into the bottom of the chamber and incubated at 37 °C. After 3 min, cells were fixed in 2.4% paraformaldehyde for 30 min. The cells were permeabilized with TritonX-100, incubated with blocking buffer for 30 min and then incubated with primary antibodies for 60 min, followed by washes and 60 min incubation with either mouse isotype specific or anti-rabbit Alexa conjugated secondary antibodies (ThermoFisher Grand Island NY). Primary antibodies used for immunostaining: pLAT 132 - rabbit polyclonal IgG from Invitrogen (Catalog # 44–224) - used at 4.38 μg/ml, pY (4G10) - mouse monoclonal IgG2B from Millipore (Catalog # 05–321) - used at 1.1 μg/ml. Secondary antibodies used for immunostaining: Alexa Fluor 647 - goat anti mouse monoclonal IgG2B from Thermo Fisher Scientific (Catalog # A21242) - used at 4 μg/ml, Alexa Fluor 568 - goat anti rabbit IgG from Thermo Fisher Scientific (Catalog #A11036) - used at 2 μg/ml.

### Imaging

Confocal imaging was performed as previously described^14^. Microscope images of fixed cells were captured using a Leica SP8 laser-scanning confocal microscope using a 63×, 1.4 numerical aperture (NA) objective (Leica Microsystems Inc, Buffalo Grove IL). 2- to 3-μm z-stacks with a spacing of 0.3 μm were taken of the area contacting the coverslip.

### Image processing

Leica AF software was used to produce images of fixed cells. Adobe PhotoShop and Illustrator (Adobe Systems Inc, San Jose CA) were used to prepare composite figures. Scale bars were cut from the original images and then were pasted in a more visible position on the final composite image.

### T cell transduction

Retroviral T cells transduction was performed as previously described^2^. Briefly, WT and CISH KO CD8^+^ T-cell blasts were obtained from lymph nodes by negative selection (Mouse CD8^+^ T cell Isolation Kit, STEM CELL, #19853) and activated with 2 μg/ml CD3, 2 μg/ml CD28 mAbs and 100 U IL2 for 1 day. The virus-containing supernatant form Plat-E packaging cells was added to 24-well non-tissue culture treated plates coated with Retronectin and anti-CD3 mAb and then centrifuged at 2500 rpm at 32 °C for 120 min. Viral particles were removed and then T-cell blasts were added in regular medium containing IL-2. After a short centrifugation, 1500 rpm for 5 min, cells were used 3 days after the transduction.

### Cell, stimulation, lysis, immunoprecipitation and Western blotting analysis

For stimulation of naïve purified CD8^+^ T cells, cells were re-suspended in RPMI alone at 50 × 10^6^ cells per 100 μL. CD8^+^ T cells were then incubated for 15 min at 4 °C with biotinylated anti-CD3ε (10 μg/mL 2C11 BD Pharmingen #553060). Cells were washed twice in RPMI, incubated with RPMI for 5 min at 37 °C, and then stimulated for the indicated time by adding streptavidin (20 μg/ml final concentration). After rapid centrifugation, cells were lysed at 4 °C for 10 min in 1% NP-40 lysis buffer (50 mM Tris pH 7.4, 150 mM NaCl, 5 mM EDTA, protease inhibitor cocktail [Roche# 11836170001], 1 mM Na_3_VO_4_, 0.1% SDS, adding 200 mM N-ethylmaleimide [NEM] for ubiquitinylation experiments). Lysates were centrifuged at 13000 rpm for 10 min at 4 °C. For immunoprecipitation, soluble material was pre-cleared with 4 μg of normal mouse IgG bound to 20 μl of Protein A/G Plus-Agarose beads (Santa Cruz Biotechnology) for 1 hr at 4 °C. The precleared samples were incubated with 4 μg of the indicated antibody (FLAG M2, Cish, GFP or PLC-γ1) previously conjugated to 30 μl Protein A/G Plus-Agarose beads. After incubation for 2 hr at 4 °C, the immunoprecipitates were washed three times with ice-cold lysis buffer.

Transduced CD8^+^ T cells, prior to simulation, were rested overnight in regular RPMI medium (supplemented with 10% fetal bovine serum, 1% penicillin-streptomycin and 50 μM β-mercaptoethanol). For ubiquitination experiments, 293 T cells were treated for 4 hrs with proteasome inhibitor MG-132 at 20 mM (Calbiochem #474790). Samples were resolved by 10% SDS–polyacrylamide gel electrophoresis or 5% gels for ubiquitinylation experiments. Blots were incubated overnight at 4 °C with the corresponding primary antibody directed against ZAP-70 (Cell Signaling Technology #2705), β-actin (Cell Signaling Technology #4970), CISH (Cell Signaling Technology #8731), PLC-γ1 (Cell Signaling Technology #2822). For immunoprecipitations, PLC-γ1 (Santa Cruz Biotechnology #sc-7290), ubiquitin (Santa Cruz Biotechnology #sc-8017), or FLAG M2 (Sigma #F3165 or anti-GFP (Roche# 11814460001) were used. GFP-HRP conjugate (Miltenyi biotec #120-002-105) and HA−Peroxidase (Sigma #H6533-1VL) were used for Western blotting. Blots were incubated with horseradish peroxidase–conjugated secondary antibodies (Millipore) for 1 hr at room temperature. ECL (enhanced chemiluminescence; SuperSignal West Pico and SuperSignal West Femto, Pierce) was used to visualize protein bands, which were quantified with ImageJ software (NIH).

### Cytokine stimulation

Cultured T cells were restimulated for 6 hr with different concentrations of anti-CD3ε (#624092, BD Pharmingen) and 2 μg/ml anti-CD28 (#553294, BD Pharmingen) with addition of protein transport inhibitor (2 μl/ml, BD GolgiStop, #51-2092KZ, BD Biosciences). For intracellular staining, cells were surface stained with fixed LIVE/DEAD (Molecular Probes), then fixed and permeabilized (Cytofix/Cytoperm Plus Fixation/Permeabilization kit, BD Biosciences) to evaluate intracellular cytokine production by flow cytometry with the following antibodies: antitumor necrosis factor-α-PE-Cy7 (1:300, #557644, eBioscience), anti-IFN-γ-Pacific blue (1:500, #487311, eBioscience) and anti-Thy1.1-APC (1:2000, (#202526, Biolegend).

### Cytometry

Cytometry was performed as previously described^15^. Cell acquisition was performed on FACSCalibur (Becton Dickinson, Franklin Lakes, NJ, USA) or LSRFortessa (BD Biosciences) flow cytometers. Data analysis was performed using FlowJo software (Tree Star, Inc., Ashland, OR, USA).

### Calcium flux measurement

Cells were incubated in RPMI containing 5 mM Indo-1 dye (#I1223, Invitrogen) and 0.5 mM Probenicid (#P8761, Sigma) for 45 min at 37 °C. Cells were washed with RPMI, re-suspended in RPMI, and incubated at 37 °C for 5 min before Ca^2+^ measurement. Biotinylated anti-CD3ε (5 μg/ml) was added, and a baseline reading was taken for 30 sec before cross-linking with streptavidin (20 μg/ml). Samples were analyzed with an LSRFortessa (BD Biosciences) with a UV laser, and data were analyzed with FlowJo software. Calcium increases were monitored as the ratio of Indo-1 (blue) and (violet) emission, and displayed as a function of time. Responses were quantified from three experiments using the area under curve (AUC) function of PrismGraph.

### Data availability

The datasets generated during and/or analyzed during the current study are available from the corresponding author on reasonable request.

### Ethical approval and informed consent

Mice used in this manuscript were maintained under pathogen-free conditions at an American Association for the Accreditation of Laboratory Animal Care-accredited facility. Mice were housed in accordance with the recommendations in the Guide for the Care and Use of Laboratory Animals of the National Institutes of Health under animal study proposals approved by the NCI-Bethesda Animal Care and Use Committee (ASP#LCMB-013).

## Results

### Cish is expressed upon TCR stimulation and regulates PLC-γ1 expression

**Figure 1:**
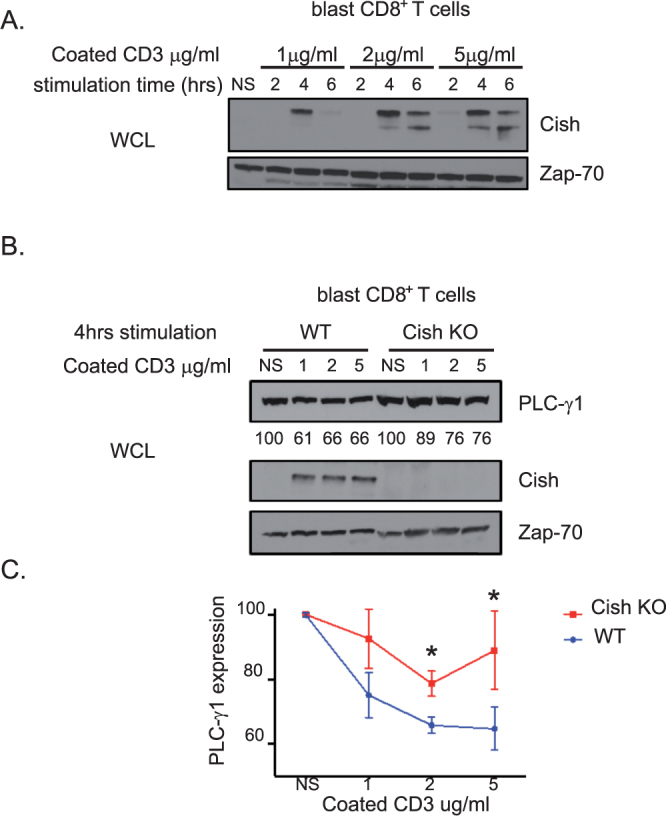
(**A**) and (**B**) Blast T cells from the indicated mice were stimulated with increasing concentration of anti-CD3 coated antibody during the indicated time. Cell lysates were analyzed by immunoblotting for Cish, PLC-γ1 and Zap-70 (Loading control). Numbers below the band indicate PLCγ1 expression quantification. (**C**) Quantification of PLC-γ1 expression from 3 experiments. Non-stimulated conditions represent 100% of PLC-γ1 expression. Bars show mean + SEM out of 3 independent experiments. Statistical significance was determined by two-tailed Student’s t test. *P ≤ 0.05. (NS = Non-stimulated).

As previously described, the Cish protein is expressed at a very low amount at steady state and increases upon TCR stimulation^2^. To more precisely study Cish expression, we stimulated blast CD8^+^ T cells for the indicated times using increasing amounts of plate-bound antibody (Fig. 1A). Interestingly, Cish expression is maximal at 4 hrs of stimulation (Fig. 1A). Indeed, for all concentrations tested, a decrease in Cish expression is detected between 4 and 6 hrs. Using α-CD3 concentrations above 2 μg/ml does not seem to increase Cish expression.

We also have showed that Cish is necessary for the ubiquitination of PLC-γ1 upon TCR activation^2^. In order to test the impact of Cish expression on PLC-γ1 degradation, we stimulated CD8^+^ T blast cells from WT or Cish KO mice using increasing amounts of plate-bound α-CD3 for 4hrs (Fig. 1B). As expected, we observed that PLC-γ1 expression decreased in the presence of Cish expression in WT CD8^+^ T cells (Fig. 1B,C) or in 293 T cells transfected with Cish and PLC-γ1 (Suppl. Figure 1). The expression of PLC-γ1 is higher in stimulated Cish KO CD8^+^ T cell compared to WT T cells, suggesting a direct effect of Cish on PLC-γ1 expression and the degradation that is dependent on TCR stimulation.

### Cish SH2 and D/BC domains are essential for PLC-γ1 ubiquitination

**Figure 2:**
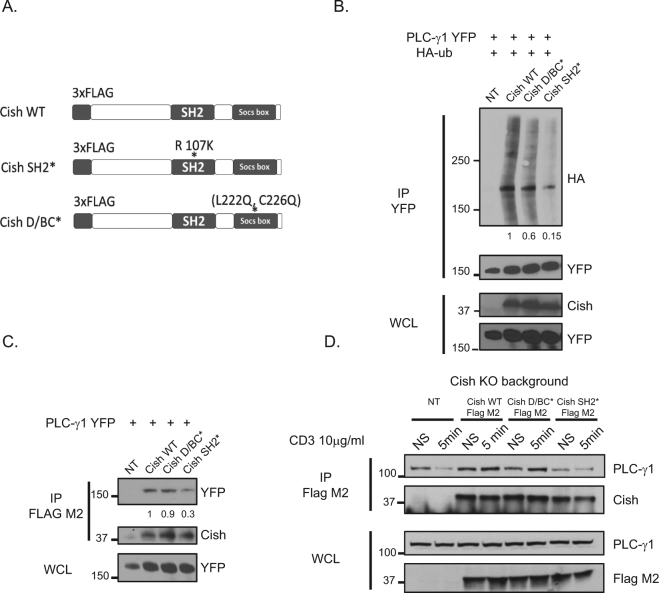
(**A**) Schematic diagram of Cish constructs used in our experiments. (**B**) 293 T cells were transfected with Tagged plasmids expressing PLC-γ1-YFP, Ubiquitin-HA, and Cish-FLAG constructs (WT, SH2* and D/BC*) where indicated, in the presence of the proteasome inhibitor, MG-132. PLC-γ1-YFP (GFP antibody) was immunoprecipitated and blotted for HA and YFP. Whole-cell lysates were blotted for Cish and Cish and YFP PLC-γ1. Numbers below the band indicate PLCγ1 ubiquitin quantification. A value of 1 was arbitrarily set for PLCγ1 ubiquitination in the presence of the Cish WT construct. (**C**) Same as in (**B**) with no proteasome inhibitor, MG-132. Cish (Flag M2) was immunoprecipitated and blotted for YFP (PLC-γ1) and Cish. Whole-cell lysates were blotted for YFP. Numbers below the band indicate PLCγ1 binding to Cish quantification, a value of 1 was arbitrarily set for PLCγ1 binding in the presence of the Cish WT construct. (**D**) Immunoprecipitation of FLAG-tagged Cish and immunoblotting of PLC-γ1 and Cish, in indicated transduced CD8^+^ T cells with and without CD3 stimulation (5 min). Whole lysates were blotted for PLC-γ1 and Flag M2.

Each SOCS protein has a conserved structure with a central SH2 domain, an amino-terminal domain of variable length and a carboxy-terminal 40-amino-acid module that is known as the SOCS box. The central SH2 domain appears critical in binding target molecules, enabling the E2-E3 complex to ubiquitinate them, but the structural and functional relationships between Cish and PLC-γ1 during CD8^+^ T cell activation remain unknown^5^. To better understand the Cish domains required for its interaction with PLC-γ1 we used mutations known to disable the SH2 domain (R107K) or to disable the SOCS box D/BC domain (L222Q, C226Q)^10,11^ (Fig. 2A). Because we have previously learned that PLC-γ1 was ubiquitinated in the presence of Cish we were able to test the effect of Cish mutant expression on PLC-γ1 ubiquitination^2^. Cultured 293 T cells were transfected with a vector expressing Cish WT, Cish SH2* or Cish D/BC* and a vector expressing PLC-γ1-YFP and/or Ubiquitin-HA. We then immunoprecipitated PLC-γ1-YFP and blotted for HA (Fig. 2B). We observed that expression of the D/BC mutant decreased PLC-γ1 ubiquitination minimally compared to expression of Cish WT. However, expression of Cish containing the SH2 mutation resulted in a major decrease in the ubiquitination of PLC-γ1 (Fig. 2B). This result is consistent with a model whereby the SH2 domain is critically important for the ubiquitination of the targeted PLC-γ1.

### The Cish SH2 domain is essential for PLC-γ1 binding

To evaluate more precisely the question of how the domains of Cish affected its physical binding to PLC-γ1, we transfected 293 T cells with vectors expressing Cish WT, Cish SH2* or Cish D/BC* in combination with PLC-γ1-YFP. We then immunoprecipitated Cish from these cells and blotted for GFP (PLC-γ1) (Fig. 2C). These data revealed an interaction of Cish WT and D/BC* with PLC-γ1 but a decreased interaction between Cish SH2* and PLC-γ1 suggesting that the SH2 domain was required for optimal interaction between Cish and PLC-γ1.

To study the Cish/ PLC-γ1 interaction in resting and stimulated T cells we transduced activated Cish KO CD8^+^ T cells with retroviral N-terminal FLAG-tagged construct expressing Cish WT, SH2* or D/BC*. After resting the transduced T cells we stimulated them with a α-CD3 antibody for five minutes and compared these cells with unstimulated T cells (Fig. 2D). To measure the physical interactions of Cish and PLC-γ1, we immunoprecipitated Cish using the Flag M2 antibody, performed electrophoresis and blotted for PLC-γ1. As observed in experiments with 293 T cells, the Cish WT protein and the D/BC* mutant interacted with PLC-γ1, but we clearly saw a decrease in PLC-γ1 binding to Cish with the SH2 mutation. We concluded that mutation of the SH2 domain of Cish, but not a mutation to the D/BC domain diminished the ability of Cish to physically interact with PLC-γ1.

### The Cish SH2 domain is essential for calcium release inhibition

**Figure 3:**
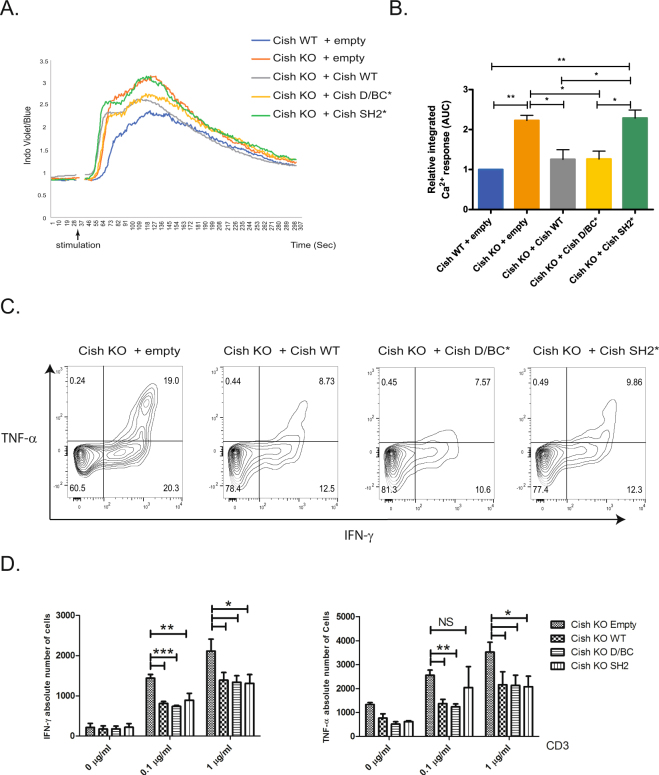
(**A**) Representative Ca^2+^ flux as assessed by fluorometric evaluation after αCD3 stimulation of indicated transduced CD8^+^ T cells. Kinetic of the ratio indo-blue to indo-violet over time shown and assessed by flow cytometry. (**B**) Calcium responses were integrated and quantified relative to Cish WT+ empty vector. Error bars represent SD from the mean (n = 3). (**C**) To evaluate cells positive for intracellular expression of TNF-α or IFN-γ, indicated transduced CD8^+^ T cells were re-stimulated with anti-CD3 coated antibody (at indicated concentration) and soluble CD28 (2 μg/ml) in presence of Golgi stop for 6 hrs. Cells were then labeled for surface Thy1.1 and intracellular TNF-α or IFN-γ. The flow cytometry analysis of Thy1.1^+^ T cell populations is shown. (**D**) Absolute cell numbers of Thy1.1^+^ T cells positive for intracellular TNF-α or IFN-γ. Bars show mean + SEM out of at least 4 independent experiments. Statistical significance was determined by two-tailed Student’s t test. *P ≤ 0.05, **P ≤ 0.01, ***P ≤ 0.001. (NS = Non-stimulated).

Once activated, PLC-γ1 hydrolyses PtdIns (4,5)P_2_ to Inositol tri-phosphate (IP_3_) and diacylglycerol (DAG), which are responsible for intracellular Ca^2+^ release and PKC activation, respectively. To evaluate the impact of CISH WT, SH2* and D/BC* expression on calcium release from CD8^+^ T cells we reconstituted Cish KO CD8^+^ T cells with retroviruses expressing either an N-terminal FLAG-Cish WT, or Cish with SH2* or D/BC* mutations (Fig. 3A,B). We examined the impact of PLC-γ1–dependent changes in cytosolic Ca^2+^ flux upon TCR stimulation in these transduced CD8^+^ T cells (Fig. 3A,B). As previously described, Cish KO CD8^+^ T cells transduced with an empty vector demonstrated an increase in Ca^2+^ release when compared to cells transduced with WT (Cish WT)^2^. This result confirmed that Cish WT constitutive expression was sufficient to inhibit Ca^2+^ release. Importantly, transduction of Cish SH2* in Cish KO CD8^+^ T cells failed to decrease Ca^2+^ release. This finding was consistent with the data shown above indicating the functional importance of the SH2 domain in binding PLC-γ1. Importantly, transduction of Cish WT and Cish D/BC* constructs in Cish KO CD8^+^ T cells had similar effects on PLC-γ1 as indicated by the Ca^2+^ decrease. These observations indicate that the SH2 domain of Cish is essential for Ca^2+^ release inhibition, most likely through PLC- γ1 binding.

### Cish SH2* and D/BC* do not impair cytokine inhibition

We already showed that Cish expression lead to a decreased release of certain cytokines such as IFN-γ and TNF-α in CD8^+^ T cells^2^. To evaluate the impact of CISH WT, SH2* and D/BC* expression on cytokine expression, we again reconstituted Cish KO CD8^+^ T cells with a retrovirus expressing N-terminal FLAG-Cish WT, SH2* or D/BC*. We then stimulated cells with plate-bound α-CD3 and soluble α-CD28 for 6 hrs, and checked by FACS for intracellular expression of IFN-γ and TNF-α (Fig. 3C,D). We observed a decrease in IFN-γ and TNF-α expression in Cish KO CD8^+^ T cells expressing either Cish WT, Cish D/BC* or Cish SH2* constructs compared to Cish KO T cells transduced with an empty vector (Fig. 3C,D). At low-dose of anti-CD3 stimulation (0.1 μg/ml), there was little difference in cytokine inhibition between cells expressing Cish WT or Cish D/BC* for production of either cytokine. However, expression of the Cish SH2* resulted in TNF-α expression not statistically different than TNF-α expression with the empty vector. The effect of the Cish SH2* protein was more restrained producing lower IFN-γ levels than cells expressing the empty vector, but higher levels than cells expressing either Cish WT or Cish D/BC*. Higher dose stimulus (1 μg/ml anti-CD3) eliminated any differences of cytokine release from cells expressing the different constructs. Thus, the Cish SH2 domain is essential for binding to PLC-γ1 and this binding is important for PLC-γ1 ubiquitination and for Ca^2+^ inhibition. However, mutation of the Cish SH2 domain has only a subtle effect on inhibition of cytokine release in the assays we performed.

### Cish WT or mutants construct expression is sufficient to inhibit PLC-γ1 clusters

In our previous study, we also observed that Cish KO CD8^+^ T cells, upon T cell activation, showed more PLC-γ1 recruitment to microclusters as detected by phosphotyrosine content compared to WT CD8^+^ T cells^2^. However we were unable to image Cish localization in those experiments due to the poor visualization of Cish protein using antibody-mediated immunofluorescence^2^. In order to localize Cish during T cell activation we generated a chimeric Cish molecule by cloning Cish WT, SH2* and D/BC* constructs into a pmTurquoise2-C1 vector^12^. We expressed these constructs and the empty pmTurquoise2-C1 vector in Jurkat T cells (Supplemental Fig. 2A). The pmTurquoise2-C1 empty vector was expressed diffusely in cytoplasm and nucleus. WT Cish mTurq was seen in the cytoplasm of both activated and unactivated cells. When transfected Jurkat cells expressing WT or mutant Cish were activated on coverslips coated with α-CD3 for 3 min, we were unable to see differences in localization of the Cish WT, SH2* and D/BC* constructs. No Cish constructs were found in any clusters upon activation (Supplemental Fig. 2B). Thus Cish is expressed in the cytoplasm and not in a specific subcellular compartment of the cell; it is not recruited to the plasma membrane. We also tried immunofluorescence staining of FLAG-tagged retroviral constructs of Cish, transduced into Cish KO activated blast T CD8^+^ cells with anti-Cish or anti Flag-Tag, but were unable to detect the Cish constructs (data not shown).

**Figure 4:**
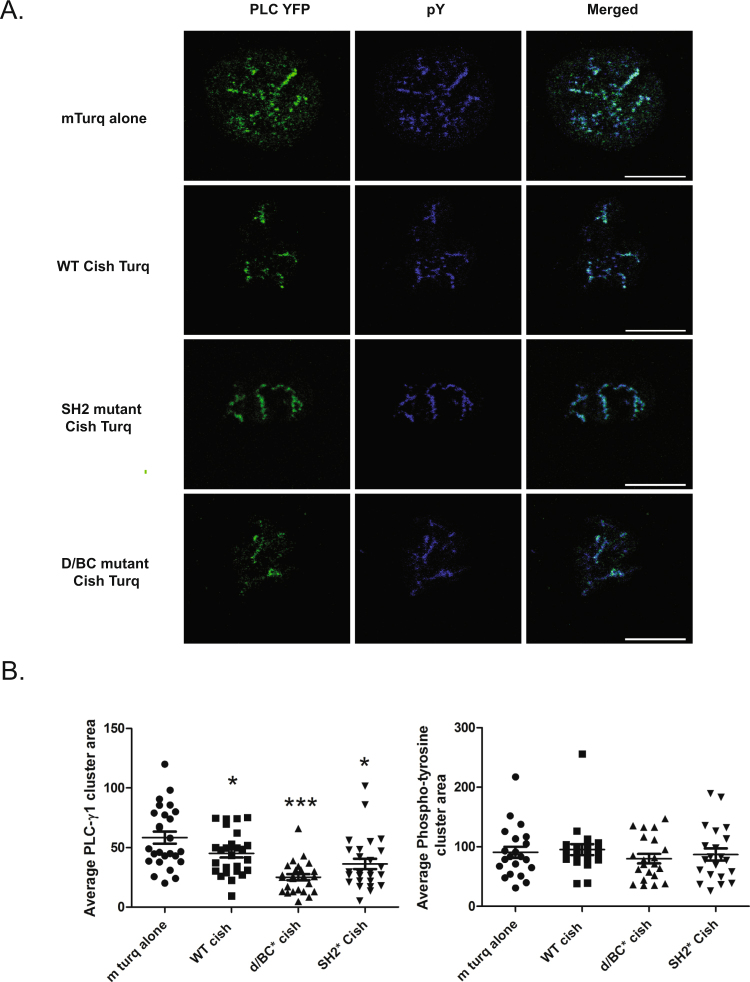
(**A**) and (**B**) Evaluation of PLC-γ1 and phosphotyrosine in TCR-induced microclusters after αCD3 stimulation in Jurkat T cells transfected with empty mTurq2-C1, Cish WT-mTurq2-C1, Cish-D/BC*-mTurq2-C1 or Cish-SH2*-mTurq2-C1 as indicated. (**A**) Representative confocal images from transfected Jurkat cells dropped on to stimulatory coverslips, fixed after three minutes, immunostained for PLC-γ1 and phosphotyrosine (pY), and the areas of the clusters were calculated (**B**). Statistical significance was determined by two-tailed Student’s t test. *P ≤ 0.05, ***P ≤ 0.001.

We then co-expressed Cish WT, SH2* and D/BC* proteins in combination with PLC-γ1-YFP in Jurkat T cells to test the effect of the various Cish constructs on microcluster formation. In this experiment, we observed fewer PLC-γ1-YFP clusters in cells transfected with Cish WT-mTurq2 compared to cells transfected with mTurq2 and stimulated with α-CD3 (Fig. 4A,B). This result confirms our previous results, and demonstrates that cytoplasmic Cish-mTurq2 is functional. Unexpectedly mutation of the SH2 or D/BC domains did not reverse PLC-γ1-YFP inhibition of cluster formation. These mutations were somewhat more efficient than Cish WT in inhibiting PLC-γ1-YFP cluster formation. However, the size of clusters detected by anti-phosphotyrosine staining was not affected by transfection of the various Cish constructs, demonstrating that the effect on PLC-γ1 was specific.

## Discussion

In an earlier publication we demonstrated that following TCR-mediated T cell activation, the SOCS family molecule, Cish, binds to PLC-γ1 leading to its degradation, thus regulating early T cell signaling and its functional consequences. The aim of our current study was to further define the structural and functional relationships between Cish and PLC-γ1 during CD8^+^ T cell activation. We show here that Cish is expressed for a very short period of time that peaks after 4 hrs of TCR stimulation. Previous data using a pulse chase assay in other cell models showed that the Cish half-life was very short (1 hr)^11^. These data indicate that Cish expression is tightly regulated in cells and especially in CD8^+^ T cells through an as yet unknown mechanism.

To study the relationship of Cish protein structure to its function we then used mutants of the Cish SH2 and SOCS-Box domains. We showed that SH2-specific binding was essential for the Cish-mediated PLC-γ1 ubiquitination and degradation. The Cish D/BC domain mutant caused a small decrease in ubiquitination. The impact of these mutations on CD8^+^ T cells functions was also tested and we showed that an intact Cish SH2 domain was essential for full inhibition of Ca^2+^ release. Expression of a Cish molecule with this mutation resulted in the same level of calcium release as was observed in Cish KO T cells without any Cish-expressing construct. Reconstitution with Cish having a D/BC mutation did not differ from reconstitution with a WT construct in the calcium assay.

In cytokine release functional assays, we reproduced our original findings that activation of Cish-deficient T cells resulted in more cytokine release than was observed from similarly activated Cish KO cells re-constituted with WT Cish protein. Levels of IFN-γ and TNF-α produced by the KO cells were significantly different than that produced by the reconstituted cells. We also showed that reconstitution of the KO cells with the Cish SH2 mutant resulted in a slight increase in cytokine expression (TNF-α greater than IFN-γ) compared to the WT while expression of the Cish D/BC mutant brought down the level of cytokine expression to that seen in cells reconstituted with WT Cish. The difference in the results of the calcium and cytokine assays may reflect the length of time of the assay. Stimulation of the T cell for the calcium release is measured in seconds and minutes, while activation for the cytokine assay is six hours. The more prolonged assay might allow sufficient function from the Cish proteins to affect cytokine production.

By genetically coupling Cish to the Turquoise fluorescent protein we were able to show that Cish was expressed predominately in the cytoplasm. We did not observe any Cish clustering at the plasma membrane or co-localization with PLC-γ1 or any other tyrosine-phosphorylated proteins upon T cell stimulation. Any effect of the Cish protein might thus occur prior to PLC-γ1 recruitment to microclusters or is so rapid at that site as to be not visible in our assay. We confirmed our previous observation that the area of microclusters containing PLC-γ1 in Cish-deficient T cells decreased in the presence of Cish, whereas the absence or presence of Cish had no effect on the area of microclusters detected using anti- phosphotyrosine antibodies. When we reconstituted the Cish KO cells with either of the SH2 or D/BC mutations, we observed a decrease in the PLC-γ1 microcluster area as we did with WT Cish reconstitution. As with the cytokine assay, the mutated protein likely had sufficient functional capacity to affect clustering of the PLC-γ1 molecule.

Immunotherapy has led to unprecedented responses in patients with advanced-stage tumors with the use of checkpoint inhibitors and adoptive cellular therapy^16,17^. Additionally, T cells can be engineered to express CAR T cell and adoptively transferred to patients^18^. All of these strategies target cell surface molecules to improve T cell anti-tumor function. We propose to identify and target intracellular molecules to improve T cell anti-tumor properties^2,19^. We previously showed that genetic deletion of the Cish molecule, an intracellular protein regulatory molecule, was an efficient strategy to improve CD8^+^ T cell function and others have shown the importance of Cish in NK anti-tumor properties^2,3^. In the current study, we performed initial structure-function studies on Cish in an attempt to discover a region of the protein that would be essential to its inhibitory properties and that could be targeted for immunotherapy development. Perhaps due to the complexity of Cish regulation, we were unable to identify an appropriate dominant negative mutant of Cish that consistently inhibited Cish in all assays. In light of this observation, we believe that a more efficient strategy to target Cish, and perhaps other intracellular proteins for immunotherapy, is to generate genetic deletion of the protein of interest in lymphocytes.

## Electronic supplementary material

supplementary information
