# Supplementary material for: The Cish SH2 domain is essential for PLC-γ1 regulation in TCR stimulated CD8+ T cells
Source: Sci Rep. 2018 Mar 28;8:5336. doi: 10.1038/s41598-018-23549-2 (PMC5871872; doi:10.1038/s41598-018-23549-2)
Supplement: Supplementary file 1 — supplementary information [file 41598_2018_23549_MOESM1_ESM.pdf]

Supplementary information :

The Cish SH2 domain is essential for PLC-γ1 regulation in TCR stimulated CD8^{+} T cells

Geoffrey Guittard[1, 3,*], Ana Dios-Esponera[1,*], Douglas C. Palmer[2,] Itoro Akpan[1], Valarie A. Barr[1], Asit Manna[1], Nicholas P. Restifo[2] and Lawrence E. Samelson[1]

Suppl.Figure.1:

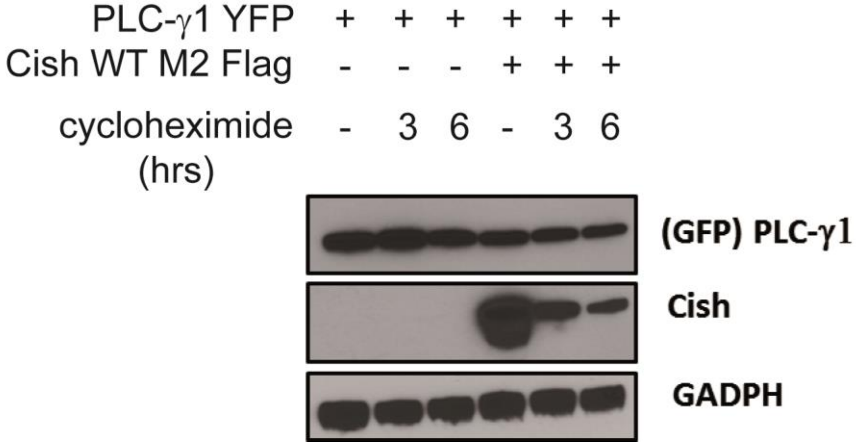

Suppl. Fig.1: 293T cells were transfected with tagged plasmids expressing PLC-γ1-YFP, and Cish WT M2-FLAG constructs as indicated. Cells were treated for the indicated time with cycloheximide 100ug/ml. After lysis cells were blotted for GFP, Cish and GADPH antibodies.

Suppl. Fig.2

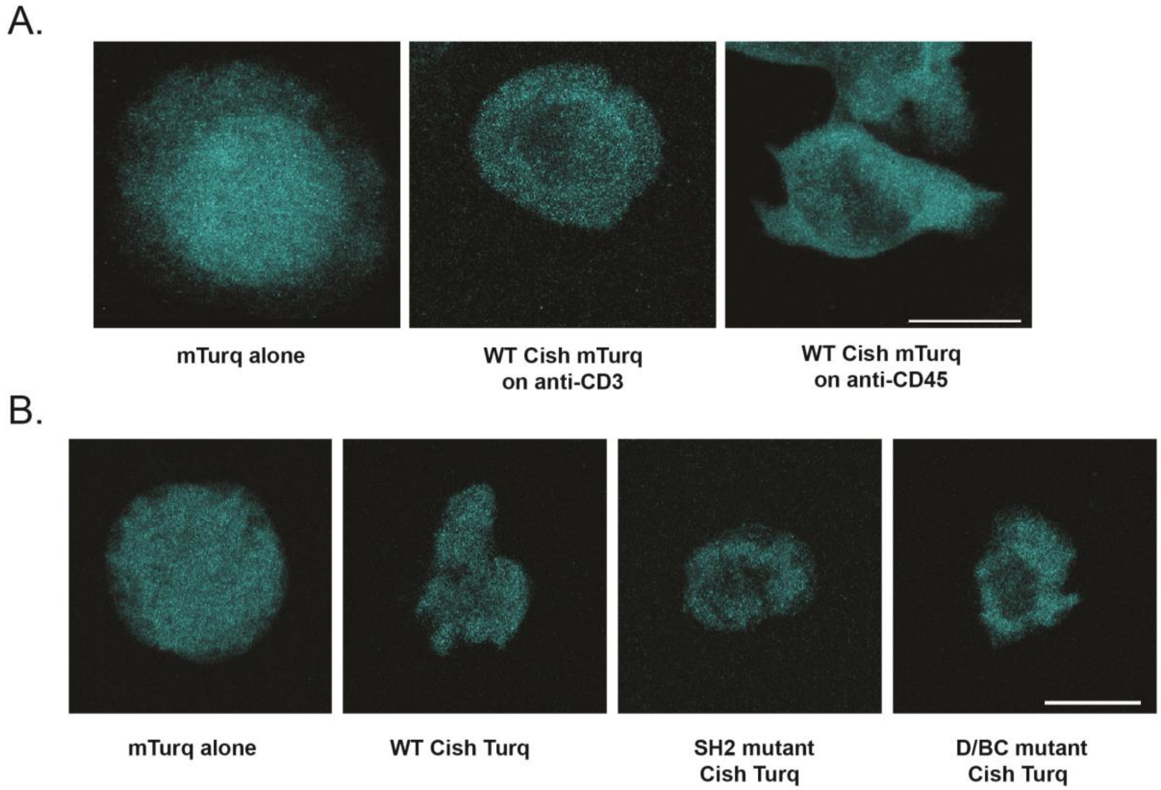

Suppl. Fig.2: A. Jurkat T cells were transfected with empty mTurq2-C1 or Cish WT-mTurq2-C1. Images were acquired of cells fixed 3 min after plating onto αCD3 or αCD45 coated coverslips. Cellular localization was evaluated in 3D renderings of the confocal z-stacks. Representative maximum projection images are shown. **B.** Jurkat T cells were transfected with empty mTurq2-C1, Cish WT-mTurq2-C1, Cish-D/BC*-mTurq2-C1 or Cish-SH2*-mTurq2-C1 as indicated. Cells were fixed after 3 min of activation.
